# The impact of repeated item development training on the prediction of medical faculty members’ item difficulty index

**DOI:** 10.1186/s12909-024-05577-x

**Published:** 2024-05-30

**Authors:** Hye Yoon Lee, So Jung Yune, Sang Yeoup Lee, Sunju Im, Bee Sung Kam

**Affiliations:** 1 Division of Humanities and Social Medicine, Pusan National University School of Korean Medicine, Yangsan, Republic of Korea; 2 Department of Medical Education, Pusan National University School of Medicine, Yangsan, Republic of Korea; 3 Family Medicine Clinic and Biomedical Research Institute, Pusan National University Yangsan Hospital, Yangsan, 50612 Republic of Korea

**Keywords:** Evaluation, Item difficulty prediction, Medical education, Faculty development training, Multiple-choice questions, Interrater reliability

## Abstract

**Background:**

Item difficulty plays a crucial role in assessing students’ understanding of the concept being tested. The difficulty of each item needs to be carefully adjusted to ensure the achievement of the evaluation’s objectives. Therefore, this study aimed to investigate whether repeated item development training for medical school faculty improves the accuracy of predicting item difficulty in multiple-choice questions.

**Methods:**

A faculty development program was implemented to enhance the prediction of each item’s difficulty index, ensure the absence of item defects, and maintain the general principles of item development. The interrater reliability between the predicted, actual, and corrected item difficulty was assessed before and after the training, using either the kappa index or the correlation coefficient, depending on the characteristics of the data. A total of 62 faculty members participated in the training. Their predictions of item difficulty were compared with the analysis results of 260 items taken by 119 fourth-year medical students in 2016 and 316 items taken by 125 fourth-year medical students in 2018.

**Results:**

Before the training, significant agreement between the predicted and actual item difficulty indices was observed for only one medical subject, Cardiology (K = 0.106, *P* = 0.021). However, after the training, significant agreement was noted for four subjects: Internal Medicine (K = 0.092, *P* = 0.015), Cardiology (K = 0.318, *P* = 0.021), Neurology (K = 0.400, *P* = 0.043), and Preventive Medicine (*r* = 0.577, *P* = 0.039). Furthermore, a significant agreement was observed between the predicted and actual difficulty indices across all subjects when analyzing the average difficulty of all items (*r* = 0.144, *P* = 0.043). Regarding the actual difficulty index by subject, neurology exceeded the desired difficulty range of 0.45–0.75 in 2016. By 2018, however, all subjects fell within this range.

**Conclusion:**

Repeated item development training, which includes predicting each item’s difficulty index, can enhance faculty members’ ability to predict and adjust item difficulty accurately. To ensure that the difficulty of the examination aligns with its intended purpose, item development training can be beneficial. Further studies on faculty development are necessary to explore these benefits more comprehensively.

## Background

Evaluation plays a vital role in determining students’ achievement of intended learning outcomes within a curriculum [1]. Boud emphasized the importance of evaluation by stating, ‘Students can escape from the effects of poor teaching, but they cannot escape the effects of poor evaluation’ [2, 3]. In the field of medical education, multiple-choice questions (MCQs) are widely used to evaluate knowledge application and offer insights into students’ academic performance [4, 5]. Valid and reliable item development is essential for effective evaluations, and subsequent item analysis is necessary to determine item quality. Classical test theory (CTT) posits that a student’s test score comprises the true and error scores. Various indices, including item difficulty, corrected-item difficulty, item discrimination, item guessing, and attractiveness of distractors, are utilized in CTT to evaluate item quality and analyze test performance [6–8]. The item difficulty index is the ratio of the number of students who choose the correct answer to the total number of students who respond to each item.

Item difficulty plays a crucial role in assessing students’ understanding of the concept being tested. Overall item difficulty, which represents the average item difficulty across all the test items, provides a general measure of the test’s difficulty level as a whole [9, 10]. The overall difficulty indices must be adjusted based on the purpose of the evaluation. For example, if an exam is used for a diagnostic evaluation to identify learning difficulties, the overall difficulty should be greater [11]. Conversely, the overall difficulty should be low if the exam is an out-of-level test designed for a few exceptional students [12]. To adjust an exam’s overall difficulty, each item needs to be developed to achieve the target difficulty index in mind. The item-author’s ability to set and accurately predict the item’s target difficulty index significantly impacts achieving the evaluation’s intended purpose. Therefore, after students complete the exam, a crucial step is to scrutinize the congruence between the actual difficulty index estimated through item analysis and the predicted difficulty index determined by the item author [13, 14].

Previous studies have shown that even short-term item development training reduces item errors or flaws and increases the number of items with an optimal difficulty index [15–17]. However, other studies have suggested the necessity of continuous or repeated faculty training sessions because short-term or single faculty training sessions are not sufficient to improve the quality of MCQ development [18–22]. A study examining the agreement between predicted and actual difficulty indices with 26 teachers reported that teachers tended to predict the difficulty index higher, indicating a tendency to perceive items as easier than they actually were [23]. Nevertheless, research on faculty development programs aimed at improving professors’ ability to adjust item difficulty as intended remains limited. Therefore, the present study aimed to assess the impact of repeated item development training for faculty members on enhancing the predictive ability of the item difficulty index in educational evaluation.

## Methods

### Study design

**Fig. 1:**
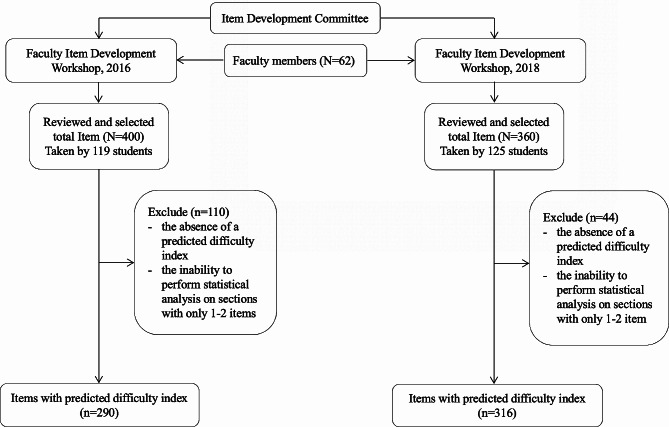
Study’s flowchart

This study compared the accuracy of item difficulty predictions estimated after the first implementation (2016) of the item development training workshop with those estimated following the workshop’s second iteration (2018). To evaluate this accuracy, the study compared the number of subjects showing significant agreement between the predicted and actual difficulty indices before and after the training. The item development training included the prediction of each item’s difficulty (Fig. 1).

### Ethical approval

As this retrospective study utilized pre-existing, de-identified data, it received an exemption from the Institutional Review Board Ethics Committee at Pusan National University Yangsan Hospital (IRB No. 2021-3).

### The examination and the examinees

The study analyzed all items from the ‘Comprehensive Clinical Evaluation’—a summative MCQ evaluation for final-year medical students, administered to fourth-year medical students at one medical school. This examination aimed to assess the students’ competencies in medical knowledge, and the results were used to determine pass/fail grading. This examination spanned various medical subjects, including Gastroenterology, Cardiology, Pulmonology, and Other Internal Medicine Subspecialties (Nephrology, Endocrinology, Allergy, Rheumatology, Infectiology, and Hemato-Oncology), as well as General Surgery, Obstetrics/Gynecology, Pediatrics, Neurology, Psychiatry, Emergency Medicine, Preventive Medicine, and Legal Medicine. 119 fourth-year students participated in the examination in 2016 and 125 in 2018.

### Item development training

In 2016 and 2018, the ‘Item Development and Modification Workshop’ for item development training was conducted by the Item Development Committee. This Workshop primarily focuses on the principles of developing MCQs. Prior to the workshop, faculty members responsible for teaching students developed newly drafted items. All item developers and reviewers were provided with data from the previous year’s examination, including the item difficulty index, discrimination index, and attractiveness of distractors.

### The item development committee

The Item Development Committee trained the item reviewers during the workshop by offering continuous feedback, enabling them to revise newly drafted items in accordance with the following principles: (1) the items must align with national exam standards; (2) the difficulty level should be within the ideal range; and (3) evaluation should focus on the application of knowledge rather than mere memorization. The Item Development Committee played a critical role by providing continuous feedback until the items met the required standards. Item reviewers also played a vital part by correcting defects and ensuring each item adhered to core item development principles.

### Item reviewers

Item reviewers appointed for each medical subject participated in the workshop held in 2016 and 2018. They received training on adjusting the item difficulty index by modifying the composition and content of the item. After completing the revision process, the reviewer submitted the predicted difficulty index for each item: In the 2016 workshop, item reviewers from each subspecialty received previously presented items and were trained to predict the difficulty index. They then compared their predictions with the actual difficulty indices, identifying and analyzing any discrepancies in the items. This training process continued in the subsequent 2018 workshop, which focused on analyzing the differences between their predicted difficulty indices and the actual difficulty indices of the 2016 examination items. After feedback from the Item Development Committee, the difficulty level for each item was initially predicted following individual review by each subspecialty. Subsequently, reviewers from each subject gathered to jointly review the newly drafted items and make the final decision on the predicted difficulty index of each item.

### Difficulty index guideline

Our school comprises a 6-year program, including a 2-year pre-medical course followed by a 4-year medical course. Based on a competency-based curriculum, our school’s program is structured into three phases. Phase 1 covers the first year and a half of pre-medical school, phase 2 extends from the subsequent period to the second year of medical school, and phase 3 includes the third and fourth years of medical school. Each phase details the expected competency standards to be achieved. The competencies are also defined for each course

**Fig. 2:**
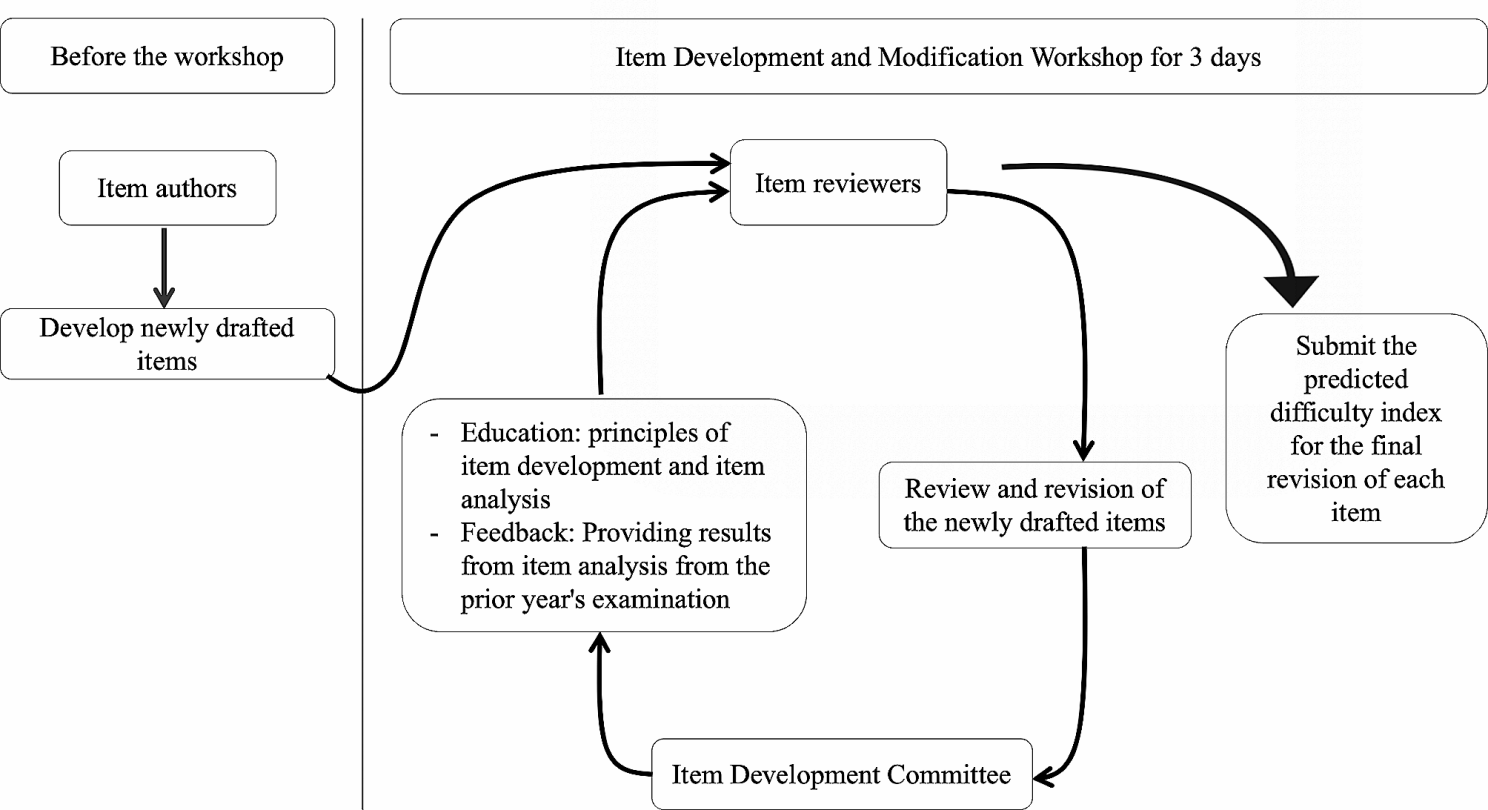
The item development and modification workshop process

During this joint review, the predicted difficulty index for each item was discussed and agreed upon before submission using the detailed expected competency standards. Through this rigorous development process, reviewers were able to refine the composition and content of candidate items for the comprehensive examination that year. We set the passing score using a norm-referenced approach, requiring a minimum of 60% correct responses across the entire test in this examination [24]. A student was considered to have passed if they scored an average of 60 points or more out of 100 on the written test. The workshop process is illustrated in Fig. 2.

### Items included in the study

In the evaluation, 400 items from 2016 to 360 items from 2018 were considered. Of these, 290 items from 2016 to 316 from 2018 were included in the analysis. Some items were excluded for reasons such as the absence of a predicted difficulty index (e.g., Obstetrics/Gynecology and Psychology in 2016) or the inability to perform statistical analysis on sections with only one or two items, for instance, medical ethics. Items specific to Emergency Medicine were included in Internal Medicine subjects (Fig. 1).

### Item analysis

The actual difficulty index for each item was calculated as the ratio of the number of correct answers to the total number of students. After an exam, faculty members received feedback on the difficulty index of the items they had predicted. The corrected item difficulty index was calculated using the following formula to exclude correct answers attributed to guessing.

$$CP=\frac{KP-1}{K-1}$$

(K: the number of distractors; P: item difficulty index; CP: corrected item difficulty index)

### Data analysis

This study’s data presentation is focused on difficulty index analysis, as the item development program prioritized predicting the difficulty level for each item. Therefore, this study did not include other item analysis metrics, such as discrimination. Descriptive statistics were used to characterize and describe the features of the sample. To assess the accuracy of difficulty prediction, we analyzed whether each subject showed significant agreement between the predicted and actual difficulty indices across all items within a subject. We compared the number of subjects showing significant agreement between the predicted and actual difficulty indices in 2016 and 2018. Cohen’s kappa and correlation analyses were performed to analyze the agreement between the predicted and actual difficulty indices and between the predicted and actual corrected difficulty indices. In the kappa analysis, difficulty levels were categorized as difficult (< 0.4), moderate (0.4 ≤ x ≤ 0.8), or easy (> 0.8) [25, 26]. If kappa analysis could not be performed because the predicted difficulty index, actual difficulty index, or actual corrected difficulty index belonged to one category, correlation analysis was conducted using either Pearson’s or Spearman’s correlation coefficient, depending on the satisfaction of normality criteria. The significance level was set at 0.05, and the data were analyzed using SPSS v. 26.0 (IBM Inc., Armonk, NY, USA).

## Results

### Faculty members

Sixty-two faculty members (40 men and 22 women) attended the item development training program in 2016 and 2018. The majority of faculty members specialized in Internal Medicine (41.9%), followed by General Surgery and Obstetrics/Gynecology (both at 14.5% each) and Pediatrics (11.3%) (see Table 1).

**Table 1:** Characteristics of workshop attendees in 2016 and 2018 (*N* = 62)

	Sex, *n* (%)			Position					
	Male	Female		Professor	Associate Professor	Assistant Professor	Clinical Teacher	Endowed-chair Professor	Visiting Professor
IM	12 (19.4)	14 (22.6)		2 (3.2)	5 (8.1)	6 (9.7)	12 (19.4)	1 (1.6)	0 (0.0)
*GE*	*2 (3.2)*	*2 (3.2)*		0 (0.0)	*2 (3.2)*	*2 (3.2)*	0 (0.0)	0 (0.0)	*0 (0.0)*
*CAR*	*3 (4.8)*	*2 (3.2)*		*1 (1.6)*	*2 (3.2)*	0 (0.0)	*2 (3.2)*	0 (0.0)	*0 (0.0)*
*PUL*	*1 (1.6)*	*3 (4.8)*		0 (0.0)	*1 (1.6)*	*1 (1.6)*	*2 (3.2)*	*1 (1.6)*	*0 (0.0)*
*Other IM*	*6 (9.7)*	*7 (11.3)*		*1 (1.6)*	0 (0.0)	*3 (4.8)*	*8 (12.9)*	0 (0.0)	*0 (0.0)*
GS	9 (14.5)	0 (0.0)		0 (0.0)	1 (1.6)	3 (4.8)	5 (8.1)	0 (0.0)	0 (0.0)
OBGY	7 (11.3)	2 (3.2)		0 (0.0)	2 (3.2)	1 (1.6)	6 (9.7)	0 (0.0)	0 (0.0)
PED	2 (3.2)	5 (8.1)		0 (0.0)	0 (0.0)	2 (3.2)	5 (8.1)	0 (0.0)	0 (0.0)
NEU	1 (1.6)	0 (0.0)		0 (0.0)	0 (0.0)	1 (1.6)	0 (0)	0 (0.0)	0 (0.0)
PSY	4 (6.5)	1 (1.6)		0 (0.0)	4 (6.5)	0 (0.0)	1 (1.6)	0 (0.0)	0 (0.0)
EMR	1 (1.6)	0 (0.0)		0 (0.0)	1 (1.6)	0 (0.0)	0 (0.0)	0 (0.0)	0 (0.0)
PM	3 (4.8)	0 (0.0)		0 (0.0)	0 (0.0)	1 (1.6)	0 (0.0)	0 (0.0)	1 (1.6)
LEG	1 (1.6)	0 (0.0)		0 (0.0)	1 (1.6)	0 (0.0)	0 (0.0)	0 (0.0)	0 (0.0)
Total	40 (64.5)	22 (35.5)		3 (4.8)	14 (22.6)	14 (22.6)	29 (46.8)	1 (1.6)	1 (1.6)

IM, Internal Medicine; GE, Gastroenterology; CAR, Cardiology; PUL, Pulmonology; Other IM includes Nephrology, Endocrinology, Allergy, Rheumatology, Infectiology, and Hemato-Oncology; GS, General Surgery; OBGY, Obstetrics/Gynecology; PED, Pediatrics; NEU, Neurology; PSY, Psychiatry; EMR, Emergency Medicine; PM, Preventive Medicine; LEG, Legal Medicine

### Descriptive analysis of the predicted and actual difficulty indices

In 2016, the predicted difficulty index for Pulmonology was the lowest, recorded at 0.59 ± 0.05, while that for Gastroenterology had the highest, at 0.81 ± 0.06. In 2018, the predicted difficulty index decreased in Neurology, at 0.46 ± 0.18, and in Psychiatry, at 0.55 ± 0.08. In contrast, there was an increase in the predicted difficulty index, ranging from 0.70 to 0.78, across several medical subjects, including Cardiology, Pulmonology, Obstetrics/Gynecology, Pediatrics, and Preventive Medicine, as detailed in Table 2.

In 2016, Other Internal Medicine Subspecialties were perceived as the easiest subjects, with the highest actual difficulty index (0.76 ± 0.26). The most challenging subject was Neurology, with the lowest actual difficulty index (0.40 ± 0.36), followed by Cardiology (0.52 ± 0.29) and Preventive Medicine (0.54 ± 0.27). In 2018, Obstetrics/Gynecology was the easiest subject, with the highest actual difficulty index (0.72 ± 0.27), while Neurology remained the most difficult (0.53 ± 0.41), followed by Preventive Medicine (0.56 ± 0.29). In 2016, the difficulty index of only one medical subject, Neurology, fell outside the desired range of 0.45 to 0.75 [27, 28]. However, by 2018, all medical subjects were within this range (see Table 2).

**Table 2:** Descriptive analysis of the predicted and actual item difficulty indices

		Before training (2016)					After training (2018)		
	No. of items^a^	Predicted P	Actual P	Actual CP		No. of items^a^	Predicted P	Actual P	Actual CP
IM	153 (145)	0.70 ± 0.12	0.62 ± 0.27	0.52 ± 0.33		145 (135)	0.71 ± 0.10	0.62 ± 0.27	0.53 ± 0.34
*GE*	*27(27)*	*0.81 ± 0.06*	*0.65 ± 0.27*	*0.56 ± 0.34*		*30 (30)*	*0.78 ± 0.07*	*0.71 ± 0.27*	*0.63 ± 0.33*
*CAR*	*26 (26)*	*0.70 ± 0.09*	*0.52 ± 0.29*	*0.40 ± 0.36*		*25 (25)*	*0.71 ± 0.10*	*0.67 ± 0.21*	*0.59 ± 0.27*
*PUL*	*24 (24)*	*0.59 ± 0.05*	*0.66 ± 0.25*	*0.58 ± 0.31*		*25 (24)*	*0.72 ± 0.07*	*0.57 ± 0.30*	*0.46 ± 0.37*
*Other IM*	*76 (68)*	*0.68 ± 0.12*	*0.76 ± 0.26*	*0.53 ± 0.32*		*65 (56)*	*0.66 ± 0.10*	*0.58 ± 0.27*	*0.48 ± 0.34*
GS	52 (48)	0.64 ± 0.06	0.63 ± 0.23	0.53 ± 0.29		41 (39)	0.60 ± 0.10	0.61 ± 0.28	0.51 ± 0.35
OBGY	52 (0)	NA	0.59 ± 0.30	0.49 ± 0.38		45 (33)	0.71 ± 0.10	0.71 ± 0.27	0.64 ± 0.33
PED	51 (51)	0.64 ± 0.08	0.61 ± 0.26	0.51 ± 0.32		45 (44)	0.70 ± 0.00	0.65 ± 0.29	0.56 ± 0.36
NEU	8 (7)	0.63 ± 0.22	0.40 ± 0.36	0.25 ± 0.45		7 (7)	0.46 ± 0.18	0.53 ± 0.41	0.41 ± 0.51
PSY	27 (0)	NA	0.71 ± 2.24	0.64 ± 0.30		25 (25)	0.55 ± 0.08	0.71 ± 0.31	0.64 ± 0.39
PM	22 (19)	0.68 ± 0.14	0.54 ± 0.27	0.43 ± 0.34		20 (13)	0.71 ± 0.13	0.56 ± 0.29	0.45 ± 0.36
LEG	20 (20)	0.65 ± 0.07	0.61 ± 0.31	0.51 ± 0.38		20 (20)	0.65 ± 0.08	0.67 ± 0.27	0.59 ± 0.34
Total	394 (290)	0.67 ± 0.11	0.61 ± 0.27	0.52 ± 0.34		348 (316)	0.67 ± 0.11	0.64 ± 0.28	0.56 ± 0.35

IM, Internal Medicine; GE, Gastroenterology; CAR, Cardiology; PUL, Pulmonology; Other IM includes Nephrology, Endocrinology, Allergy, Rheumatology, Infectiology, and Hemato-Oncology; GS, General Surgery; OBGY, Obstetrics/Gynecology; PED, Pediatrics; NEU, Neurology; PSY, Psychiatry; PM, Preventive Medicine; LEG, Legal Medicine; P, difficulty index; CP, corrected difficulty index; NA, not applicable, as no item had a predicted P

^a^Number of items with predicted P values in parentheses

### Agreements between the predicted and actual item difficulty indices

In 2016, only Cardiology showed a statistically significant agreement (K = 0.106, *P* = 0.021) between the predicted and the actual corrected difficulty indices, with no such agreement in other medical subjects and the total items. However, in 2018, significant agreements were found for four subjects: Neurology (predicted and actual difficulty index, K = 0.400, *P* = 0.043), Internal Medicine (predicted and actual difficulty index, K = 0.092, *P* = 0.015; predicted and actual corrected difficulty index, K = 0.070, *P* = 0.013), Cardiology (predicted and actual difficulty index, K = 0.318, *P* = 0.021; predicted and actual corrected difficulty index, K = 0.179, *P* = 0.037), and Preventive Medicine (predicted and actual difficulty index, K = 0.577, *P* = 0.039; predicted and actual corrected difficulty index, K = 0.577, *P* = 0.039). Furthermore, the total items analysis showed a significant agreement between the predicted and actual difficulty indices (Pearson’s *r* = 0.144, *P* = 0.043), and between the predicted and actual corrected difficulty indices (Pearson’s *r* = 0.144, *P* = 0.043), as detailed in Table 3.

**Table 3:** Agreements between predicted and actual item difficulty indices

	2016						2018				
	No. of items^a^	Predicted P and Actual P		Predicted P and Actual CP			No. of items^a^	Predicted P and Actual P		Predicted P and Actual CP	
		K or r	*P*	K or r	*P*			K or r	*P*	K or r	*P*
IM	145	0.040	0.276	0.040	0.220		135	0.092	0.015	0.070	0.013
*GE*	*27*	*0.063*	*0.627*	*0.164*	*0.177*		*30*	*0.196*	*0.102*	*0.179*	*0.049*
*CAR*	*26*	*0.098*	*0.085*	*0.106*	*0.021*		*25*	*0.318*	*0.021*	*0.179*	*0.037*
*PUL*	*24*	*0.199* ^*c*^	*0.103*	*0.199* ^*c*^	*0.103*		*24*	*0.302* ^*c*^	*0.152*	*0.302* ^*c*^	*0.152*
*Other IM*	*68*	*0.001*	*0.912*	*-0.009*	*0.828*		*56*	*-0.024*	*0.444*	*-0.028*	*0.342*
GS	48	0.171^b^	0.245	0.171^b^	0.245		39	0.011	0.814	0.010	0.760
OBGY	0	NA	NA	NA	NA		33	-0.113	0.084	-0.108	0.073
PED	51	-0.081^b^	0.570	-0.081^b^	0.570		44	ND	ND	ND	ND
NEU	7	-0.273	0.115	-0.273	0.115		7	0.400	0.043	0.054	0.659
PSY	0	NA	NA	NA	NA		25	0.121^c^	0.564	0.121^c^	0.564
PM	19	-0.035	0.706	-0.032	0.673		13	0.577^c^	0.039	0.577^c^	0.039
LEG	20	0.124^c^	0.604	0.124^b^	0.604		20	-0.099^c^	0.677	-0.099^c^	0.677
Total items	290	0.069^b^	0.238	0.069^a^	0.238		316	0.144^b^	0.043	0.144^b^	0.043

IM, Internal Medicine; GE, Gastroenterology; CAR, Cardiology; PUL, Pulmonology; Other IM includes Nephrology, Endocrinology, Allergy, Rheumatology, Infectiology, and Hemato-Oncology; GS, General Surgery; OBGY, Obstetrics/Gynecology; PED, Pediatrics; NEU, Neurology; PSY, Psychiatry; PM, Preventive Medicine; LEG, Legal Medicine; P, difficulty index; CP, corrected difficulty index; NA, not applicable, as no item had a predicted P; ND, not conducted, since the predicted P was a constant value

*Note *^a^Number of items with a predicted item difficulty index. Data are presented as Kappa (K), Pearson’s correlation coefficient (r)^b^, or Spearman’s correlation coefficient (r)^c^

## Discussion

This study investigated whether repeated item development training for faculty members, including item difficulty estimation, enhances their ability to predict the item difficulty index. In the second workshop, the number of items submitted with predicted difficulty indices increased by 8.9%. Notably, the obstetrics/gynecology and psychiatry subjects, which had no submissions in 2016, also submitted predicted difficulty indices. After the implementation of this training, there was an increase in the number of subjects with an average difficulty index that fell within the desired difficulty index range, as well as an increase in the number of medical subjects and an improvement in total items showing agreement between the predicted and the actual difficulty indices. These results indicate that faculty members have the ability to predict and adjust item difficulty, a skill that can be enhanced contingent upon providing appropriate systematic and efficacious training.

Previous studies have highlighted the importance of education and training for authors and reviewers to develop items that align with desirable difficulty levels. Essential training goals include reducing item-writing flaws, accurately understanding a student’s level, and effectively delivering planned class content [15–21, 29, 30]. Studies have reported that training faculty in item development, emphasizing the cover-the-options rule, item suitability for student performance, precise and affirmative sentences, avoidance of cues, reconfirmation of correct answers, avoidance of implausible distractors, and refraining from the use of “all of the above” or “none” options can reduce item-writing flaws [8, 15, 17]. In the workshop conducted in this study, faculty members were trained as described above. A study found teachers tend to underestimate the performance of borderline (low achieving) students while overestimating that of others. This discrepancy contributes to a low agreement between predicted and actual difficulty levels for the entire student population [23]. However, our study provided faculty members with the actual difficulty index for each item and the response rate for each option, potentially improving their insight into students’ abilities. Additionally, before the start of the course sessions, all the faculty members were requested to develop items, and among them, those who participated in the 2016 and 2018 workshops reviewed and revised these items. This process can remind faculty of essential learning content and help them convey it effectively to students in subsequent classes.

Other previous studies have also demonstrated that faculty members trained in MCQ writing exhibit significantly fewer item-writing flaws [15–18, 21]. The item-flaw rate among trained faculty was 34%, compared to 76% for untrained faculty [16]. Even a one-hour training session markedly improved MCQ item-writing quality in a dental school [15]. Additionally, the impact of item-writing training was more pronounced among junior faculty than among senior faculty [17]. However, Sezari et al. [18] highlighted the need for repetitive training, noting that faculty knowledge and skills showed short-term improvement following even a one-day MCQ workshop. A longitudinal faculty development program has also demonstrated significant improvements in the faculty’s quality of MCQ item-writing skills over successive academic years [21]. The longitudinal faculty development program has shown significant enhancements in MCQ item-writing skills over successive academic years [21], with decreases in the proportion of poorly discriminating items (from 12.2 to 8.4%, *P* = 0.047) and item-writing flaws (from 8.5 to 3.0%, *P* = 0.001) and increases in the proportion of items with difficulty indices of 0.2 to 0.7 (from 19.5 to 30.3%, *P* = 0.0001) and attractive distractors (from 15.0 to 29.7%, *P* = 0.0001) [21]. Our study showed that in 2016, Neurology, a medical subject, exceeded the desired difficulty range of 0.45–0.75. By 2018, however, the difficulty levels of all medical subjects had adjusted to fall within this range. In another study using a self-checklist system for item authors to manage the quality of items in mock exams conducted by national medical schools twice a year, the difficulty index was maintained consistently at 0.6–0.7 for six years [31]. Previous studies using item development workshops or a self-checklist system have shown that the difficulty index can be adjusted to the appropriate level through training [15–21, 31], which is consistent with our research findings. However, unlike the present study, those previous studies did not compare the predicted difficulty index of the items to their actual difficulty index after evaluation.

Kiessling et al. [32] assessed the predictability of MCQ item difficulty using a five-point Likert scale by item authors and reviewers for undergraduate medical students’ end-of-term examinations. They found that factors such as attending a workshop on MCQ construction, receiving feedback on the actual P from previous examinations, and having experience in item reviewing significantly increased the accuracy of the authors’ difficulty predictions. As expected, the difficulty estimates made by the item authors and reviewers were similar. Our study supports the findings of Kiessling et al. [32] and extends them, offering more advanced insights. In this study, we observed an increase in the number of medical subjects with statistically significant agreement between the predicted and the actual corrected difficulty indices, indicating enhanced precision in faculty members’ estimations of difficulty. Following the administration of future exams, faculty members involved in item development training received feedback on the actual difficulty indices of the test items they had predicted. This feedback on item analysis results from previous exams can help faculty members understand the students’ level and adjust the difficulty level of the following exam [33, 34]. Furthermore, predicting and submitting difficulty indices was more than obtaining feedback on item analysis; it encouraged faculty members to engage in cognitive reflection, actively considering the item’s difficulty.

Evaluation plays an essential role in communication between students and teachers, thereby offering students opportunities for self-reflection and motivating learning [1–3]. Furthermore, the evaluation provides information about the curriculum and level of the students. Generally, items that are too easy or too difficult (difficulty index > 0.95 or < 0.30) can demotivate students and fail to reflect their overall performance. Appropriate levels of difficulty foster enhanced learning and act as a trigger for overcoming conceptual obstacles encountered throughout the learning process [35]. Quality indices, including difficulty, discrimination, reliability, and validity, must be appropriately set to meet the ultimate objectives of the evaluation, necessitating a diverse range of item difficulties [31]. To develop high-quality items, the item development workshop in the present study aimed to set items at appropriate difficulty levels by predicting difficulty indices, promoting even coverage across diverse fields and learning subjects, and constructing items in a ‘problem-solving’ format to achieve high discrimination. Additionally, feedback on the level of difficulty, discrimination, and attractiveness of incorrect options was consistently provided in every evaluation.

This study’s analysis did not include indicators other than the difficulty index, such as the discrimination index. However, examination validity relies on both appropriate difficulty and discrimination indices. The discrimination index tends to increase as the difficulty index decreases, and vice versa [36, 37]. Although lowering the difficulty index might appear to be a quick way to enhance discrimination, this strategy is generally inadvisable. It is important to maintain items at an appropriate difficulty level that reflects the intent and purpose of the examination. To ensure alignment between predicted and actual item difficulty, item authors need to understand student characteristics and how item difficulty can vary depending on the intended learning objectives. Educational experience also plays a key role in this understanding. Therefore, receiving and carefully reviewing feedback on item analysis results after test administration supports refining future examinations.

This study has several limitations. First, generalizing the effects of item development training conducted at a single medical school is challenging. Second, there were differences in the characteristics of students who took exams in 2016 and 2018, making it impossible to dismiss the influence of external factors beyond the faculty’s item development training on the outcomes. Third, this study focused on predicting difficulty indices within the context of CTT. However, faculty development includes not only item difficulty index but also other item development principles, such as item discrimination index, to ensure high-quality examination; all items in the exam had diverse levels of difficulty, and in the process, readjustments for some items were made to eliminate item-writing flaws that hinder discrimination as these flaws can impact other metrics too [8]. After completing each item development workshop, the items were not modified further. Starting in the 2016 workshop, faculty were required to submit predicted difficulty indices for each item. Initially, some faculty members found it challenging to predict difficulty. However, repeated training on difficulty prediction led to a substantial rise in the number of items submitted with predicted difficulty indices by the 2018 workshop. Despite these limitations, to the best of our knowledge, this study is the first to examine the impact of repeated item development training on improving medical faculty members’ ability to predict MCQ item difficulty. Furthermore, we highlighted the importance of developing problem-solving items with high discrimination, providing education to item authors, and discussing essential considerations in item development. The workshop also aimed to enhance item quality by revising items to remove writing flaws and reduce cues that impair discrimination.

## Conclusions

In summary, the results of this study suggest that item development training, which includes predicting the difficulty index of each item, can enhance faculty members’ ability to accurately predict and adjust item difficulty in medical **assessment**. The implementation of this training significantly increased the number of items within the desired difficulty range and increased the number of medical subjects with the predicted difficulty index aligning with actual difficulty index. To ensure that the difficulty of the examination aligns with its intended purpose, item development training can be beneficial. Further studies on faculty development are necessary to explore these benefits more comprehensively.

## Data Availability

The datasets used and analyzed during the current study are available from the corresponding author upon reasonable request.

## Abbreviations

MCQs: Multiple-choice questions
CTT: Classical test theory
